# Efficacy of Transcranial Direct Current Stimulation Over Dorsolateral Prefrontal Cortex in Patients With Minimally Conscious State

**DOI:** 10.3389/fneur.2022.821286

**Published:** 2022-02-18

**Authors:** Yuan Peng, Jingpu Zhao, Xiao Lu, Juntao Dong, Shunxi Zhang, Jin Zhang, Huihua Liu, Xiuyuan Zheng, Xin Wang, Yue Lan, Tiebin Yan

**Affiliations:** ^1^Department of Rehabilitation Medicine, Guangzhou First People's Hospital, School of Medicine, South China University of Technology, Guangzhou, China; ^2^Department of Rehabilitation Medicine, Sun Yat-sen Memorial Hospital, Sun Yat-sen University, Guangzhou, China; ^3^Department of Rehabilitation, Shenzhen Second People's Hospital, The First Affiliated Hospital of Shenzhen University, Shenzhen, China; ^4^Department of Rehabilitation Medicine, Guangdong 999 Brain Hospital, Guangzhou, China; ^5^Huazhong University of Science and Technology Union Shenzhen Hospital, Shenzhen, China; ^6^Department of Rehabilitation Medicine, Clinical Medical College, Yangzhou University, Yangzhou, China

**Keywords:** minimally conscious state, transcranial direct current stimulation, coma recovery scale-revised score, functional connectivity, left dorsolateral prefrontal cortex

## Abstract

**Background:**

The treatment of patients in a minimally conscious state (MCS) remains challenging. Transcranial direct current stimulation (tDCS) is a non-invasive therapeutic method in treating neurologic diseases by regulating the cortical excitability. The aim is to investigate the effect of tDCS in patients with MCS in this study.

**Methods:**

Eleven patients in MCS were enrolled in the study. All the patients received 5 daily sessions of 20-min sham tDCS, followed by 10 sessions of 20-min real tDCS. The anodal electrode and cathodal electrodes were placed over the left dorsolateral prefrontal cortex (DLPFC) and the right eyebrow, respectively. Assessment of Coma Recovery Scale-Revised (CRS-R) scores and resting-state functional MRI (rs-fMRI) scans was conducted three times in each patient: before tDCS (baseline, T0), post-sham tDCS at week 1 (T1), and post-real tDCS at week 2 (T2). The whole-brain functional connectivity (FC) was obtained by bilaterally computing FC from six seed regions: precuneus, middle frontal gyrus, supplemental motor area, angular gyrus, superior temporal gyrus, and occipital lobe. One-way repeated measure ANOVA was used to compare the differences of CRS-R scores and FC at T0, T1, and T2. The false discovery rate correction of *p* < 0.001 was adopted for controlling multiple comparisons in FC analysis.

**Results:**

Five patients with MCS showed obvious clinical improvement represented by increased CRS-R scores post- 2-week real tDCS. The CRS-R scores did not change post- 1-week sham treatment. No side effects were reported during the study. The FC of the bilateral supplementary motor area, right angular gyrus, and right superior temporal gyrus were significantly enhanced after 2-week real tDCS compared with that after 1-week sham-tDCS. In addition, FC of bilateral occipital lobe and right precuneus were significantly enhanced post- 2-week real tDCS compared with the baseline.

**Conclusion:**

Our findings indicated that tDCS over DLPFC could serve as a potentially effective therapy for improving the consciousness state in patients with MCS. The FC in rs-fMRI can be modulated by tDCS at both the stimulation site (left DLPFC) and the distant regions.

## Introduction

Minimally conscious state (MCS) is a disorder of consciousness (DoC), showing a partial retention of consciousness of self or environment, usually caused by neurological diseases, such as brain trauma, post-anoxic encephalopathy, and cerebrovascular accident tumor (1, 2). As MCS has the potential of continuous improvement and could attain favorable outcomes, the primary therapeutic goal of MCS is to promote arousal and awareness by pharmacological and non-pharmacological treatments (3). Even though many different pharmacological interventions have been used to date, the evidence for their effectiveness is limited (2). Therefore, research on non-pharmacological strategies for improving arousal outcome in MCS is highly warranted (2, 4). Although traditional non-pharmacological strategies, such as physical therapy and sensory stimulation, could promote arousal, to some extent, they cannot promote the recovery of the damaged consciousness-related neural networks in MCS (5). With growing understanding of the neural network changes of consciousness disorders, the novel rehabilitation method, which directly modulates the cortical excitability and neural network, provides new opportunities for the treatment of MCS (6).

Transcranial direct current stimulation (tDCS) is a non-invasive stimulation technique that can modulate cortical excitability (7). It has been used in treating neurological and psychiatric diseases, including Alzheimer's disease, stroke, and Parkinson's disease (8–10). Anodal stimulation of tDCS in left dorsolateral prefrontal cortex (DLPFC) has been shown to improve cognitive abilities in healthy individuals as well as patients with stroke (5). However, few studies have focused on the effect of tDCS in patients with consciousness disorders, such as vegetative state and MCS, in clinical trials (11, 12). The potential mechanism of such treatment may be related to the critical role of long-range fronto-parietal connections in consciousness (13). Nevertheless, its specific mechanism on neuroplasticity is still unclear, which limits the clinical application of tDCS in MCS (4).

Resting-state functional MRI (rs-fMRI) is a non-invasive and powerful tool to investigate the brain activation even without the proactive cooperation of the subjects in MCS (14). The resting-state functional connectivity (FC) in the brain is a stable and useful index to identify the functional interaction between brain regions, which has been used to understand the neural mechanisms in MCS (15). Several previous studies have demonstrated that the FC changes over time in the resting state were significantly correlated with the level of conscious state, which was often indexed by a subjective behavioral assessment, such as Coma Recovery Scale-Revised (CRS-R) (16, 17). Even though some studies reported significant changes in FC caused by tDCS in stroke and Parkinson's disease, it is unclear to what extent the functional connectivity alterations would occur in MCS by administering tDCS (18, 19).

Based on the hypothesis that tDCS could improve the conscious state by regulating brain activity and modulating brain network in MCS, we conducted the current study to assess the effects of tDCS stimulation over DLPFC on clinical status in patients with MCS, accompanied by exploring the underline mechanism through detecting the functional connectivity changes between specific regions by rs-fMRI.

## Methods

### Participants

The participants were all recruited from the hospital named “999 Brain Hospital of Guangdong Province” between September 2017 and October 2018. All enrolled participants must meet the inclusion criteria: (1) age over 18; (2) meet the criteria for minimally conscious state according to Aspen Neurobehavioral Conference Workgroup (20), with a stable level of responsiveness assessed by 3 times of the Chinese version of coma recovery scale-revised (CRS-R) in 2 weeks before enrollment; (3) time from the onset ≥ 1 month; (4) no history of contraindication to MRI; (5) no history of sedative use; and (6) no other neuromodulation therapy (e.g., transcranial magnetic stimulation) performed. The conventional physical therapy of the patient was continued during the whole experimental period. The study was approved by the Institutional Review Board in 999 Brain Hospital of Guangdong Province. Informed consent was obtained from legal representative of patients.

### Study Overview

**Figure 1:**
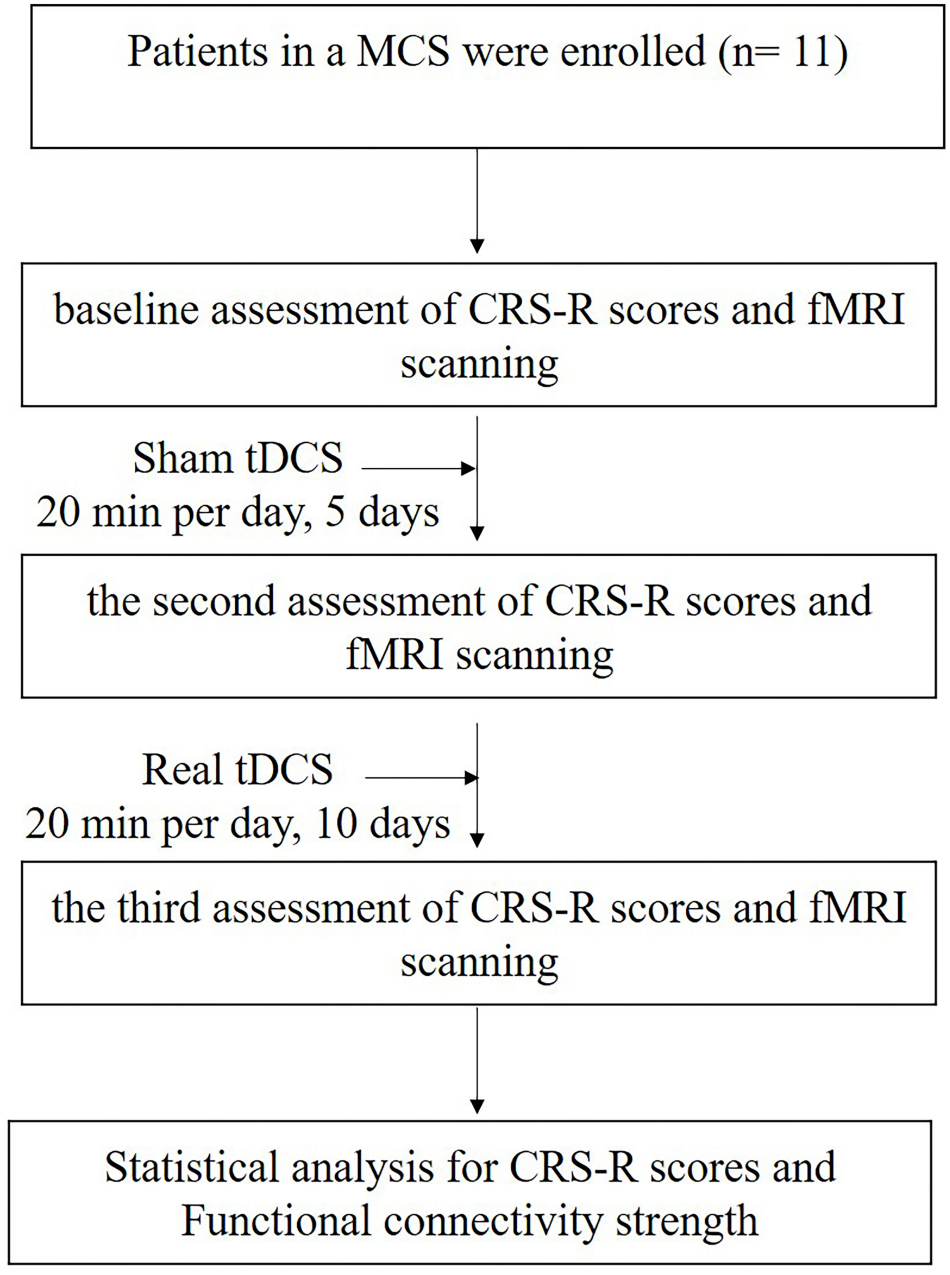
A flow chart illustrating the process for the study. MCS, minimally conscious state; tDCS, transcranial direct current stimulation; CRS-R, Coma Recovery Scale-Revised.

The study consisted of three phases. The first phase was baseline assessment of CRS-R and fMRI scanning followed by a 1-week of the sham tDCS phase, which involved a daily sham tDCS for 5 days. Then, all the patients had the second assessment of CRS-R and fMRI scanning. After that, a 2-week real tDCS treatment phase was performed for 10 daily sessions. Last, all the patients performed the third assessment of CRS-R and fMRI scanning. The patients were required not to change their original medication regimen during the study. The research protocol is illustrated in Figure 1.

### Stimulation Protocol

The tDCS was applied by using the DC stimulator (Soterix 1X1, Model 1300A, USA), which is a battery-powered constant-current stimulator. The transcranial direct current was delivered *via* saline-soaked surface sponge electrodes (7 × 5 cm). The anode was placed over the left DLPFC, which is located at F3 from the 10–20 international electroencephalogram system. The cathode was placed on the right supraorbital area (Fp2). The same electrode placement was used for both sham and real tDCS stimulations. For sham tDCS, the current was applied up to 2 mA with a ramp-in and ramp-out phase of each 30 s, and then the current was kept at 0 mA for the rest of the 20 min to build up a placebo mode. The 1-week sham tDCS was delivered once a day, for 5 weekdays. For the real tDCS, the stimulating current raised to 2 mA within 30 s and lasted for 20 min. The 2-week real tDCS was delivered once a day, for 10 weekdays.

### Behavioral Assessments

The consciousness level of each participant was evaluated right prior to fMRI scan using one CRS-R assessment. The CRS-R is a common scale for the assessment of consciousness, which was used to differentiate vegetative state (VS) from MCS and identify patients who have emerged from MCS (21). The CRS-R is comprised of six subscales, including auditory, visual, motor, oromotor, communication, and arousal function organized in 29 hierarchically items to reflect the level of consciousness and to track behavioral recovery, and each item has specific scoring criteria (22, 23). In addition, adverse events related with the stimulation like allergies and redness of the skin were also recorded in the study.

### MRI Data Acquisition

Structural MRI and rs-fMRI were conducted with a 3T GE MR750 scanner equipped with a standard bird cage head coil. All the MRI data were collected within 24 h after enrollment of the participants; the 1-week sham-tDCS and the 2-week real-tDCS stimulation, respectively. We collected the high-resolution T1-weighted images from all the patients to reconstruct their individual structural brain anatomy. The parameters were as follows: repetition time (TR) = 8.2 ms, echo time (TE) = 3.2 ms, flip angle (FA) = 15°, flip angle (T1) = 450 ms, field of view (FOV) = 240 × 240 mm, slice thickness = 1 mm, voxel size = 0.93 mm × 0.93 mm × 1.00 mm^3^, matrix = 256 × 256, number of layers = 164. We adopted an echo planar imaging sequence to gain the resting state fMRI data (TR = 2,000 ms, TE = 30 ms, FOV = 240 × 240 mm, FA = 78°, voxel size = 3.75 mm × 3.75 mm × 1.00 mm^3^), repetitive times = 240.

### Functional Connectivity Analysis

The rs-fMRI data were analyzed using the Statistical Parametric Mapping (SPM8), with a MATLAB toolbox (R2013a) named Data Processing Assistant for Resting-State fMRI (24, 25). The following steps were done automatically by the method: convert DICOM files into NFTI images, slice-timing correction, head motion realignment, normalization into Montreal Neurological Institute (MNI-152) space, smoothing with 6-mm full width at half maximum Gaussian kernel, remove a linear tread to diminish the influence of covariates, and image filtering (0.01–0.08 Hz) for getting rid of the high-frequency signal.

The Resting-State fMRI Data Analysis Toolkit was then used for the computation of brain functional connectivity (FC). We computed the whole-brain FC by analyzing the FC from six regions of interest (ROIs) bilaterally: occipital lobe, precuneus, supplementary motor area, angular gyrus, superior temporal gyrus, and middle frontal gyrus. FC refers to the sum of the energy and energy connections between each voxel and all other voxels in the entire brain (17). Functional connectivity reflects effective correlations between different regions in neuronal information processing. The ROI-based correlation was used to do the FC analysis in the present study. The larger FC of the selected ROI has, the closer its inter-relationship with other brain regions is. Peak coordinates of ROIs were selected according to pieces of literature, in which 10-mm-radius and 4-mm-radius spheres around peak x, y, and z coordinates were delineated for cortical areas and subcortical structures, respectively (17, 26). The peak x, y, and z coordinates of each ROI are listed in Supplementary Table 1.

### Statistical Analysis

Statistical analysis was performed using SPSS 22.0 (IBM Corp., Armonk, NY, USA). Data are described as means ± standard deviations (SDs). A one-way repeated-measures ANOVA was performed to compare the difference of a behavioral outcome, including CRS-R total and subscales scores between the three time points (T0, T1, and T2), in which the least significant difference test was performed for multiple comparison corrections. A statistical significance was set at *p* < 0.05. To compare the differences of FC between the time points (T0, T1, and T2), ANOVA analysis was performed, and false discovery rate (FDR) correction of *p* < 0.001 was used for controlling multiple comparisons.

## Results

### Baseline Characteristic

Eleven patients with MCS were enrolled, including 10 patients with traumatic brain injury and one patient with hemorrhagic stroke. The baseline features of the patients include age, gender, and time from the onset, which are presented in Table 1. The average time from the onset was 3.36 ± 1.36 months. Seven of the 11 patients with MCS were caused by diffuse axonal injury. The tDCS stimulations were well tolerated by all the patients with no significant adverse events linked to the stimulation.

**Table 1:** Demographic and clinical characteristics of the 11 patients with MCS.

**Patient**	**Age (year)**	**Gender**	**Etiology**	**Lesion location**	**Time from onset (month)**	**CRS-R scores**		
						**T0**	**T1**	**T2**
P1	19	Male	TBI	Bilateral frontal parietal lobes	1	10	10	16
P2	40	Male	TBI	Diffuse axonal injury	2	14	14	14
P3	37	Male	TBI	Diffuse axonal injury	3	12	12	17
P4	35	Male	TBI	Diffuse axonal injury	3	9	9	14
P5	62	Female	TBI	Right frontotemporal lobes	3	15	15	15
P6	37	Male	Stroke	Left frontotemporal and parietal lobes	3	9	9	16
P7	55	Male	TBI	Diffuse axonal injury	4	13	13	13
P8	51	Male	TBI	Diffuse axonal injury	4	13	13	13
P9	31	Male	TBI	Right temporal lobe and right basal ganglia	5	10	10	16
P10	44	Male	TBI	Diffuse axonal injury	3	13	13	13
P11	62	Female	TBI	Diffuse axonal injury	6	14	14	14

### Behavioral Outcome

The CRS-R scores of 11 patients at the baseline (T0), post-1-week sham tDCS (T1), and post-2-week real tDCS (T2) are also shown in Table 1. The CRS-R subscales scores at each assessed time point are provided in Supplementary Table 2. The CR-R scores did not change in post-1-week sham tDCS compared to the baseline. However, the CRS-R scores were significantly improved post-2-week real tDCS compared with those at the baseline and post-1-week sham tDCS. Importantly, five out of the 11 patients showed an obvious improvement of CRS-R scores. As to the CRS-R sub-domains, the changes were significant in the auditory function (*p* = 0.03) and motor function (*p* = 0.02) of post-2-week real tDCS compared with those post-1-week sham tDCS. In contrast, the scores of visual, oromotor, communication, and arousal functions were not significantly different with post-2-week real tDCS (Table 2).

**Table 2:** CRS-R total and subscales scores of the 11 patients with minimally conscious state (MCS) at each time point.

	**T0**	**T1**	**T2**	**F**	***p-*** **value**
CRS-R	12.00 ± 2.14	12.00 ± 2.14	14.63 ± 1.43	6.79	0.004
Auditory	2.27 ± 0.65	2.27 ± 0.65	2.91 ± 0.54	3.95	0.03
Visual	1.73 ± 0.47	1.73 ± 0.47	2.09 ± 0.54	2.00	0.15
Motor	3.55 ± 1.37	3.55 ± 1.37	4.82 ± 0.60	4.34	0.02
Oromotor	1.36 ± 0.50	1.36 ± 0.50	1.55 ± 0.52	0.46	0.63
Communication	1.00 ± 0.00	1.00 ± 0.00	1.09 ± 0.30	1.00	0.38
Arousal	2.09 ± 0.30	2.09 ± 0.30	2.18 ± 0.40	0.26	0.77

### FC Data Reflected in rs-fMRI

**Figure 2:**
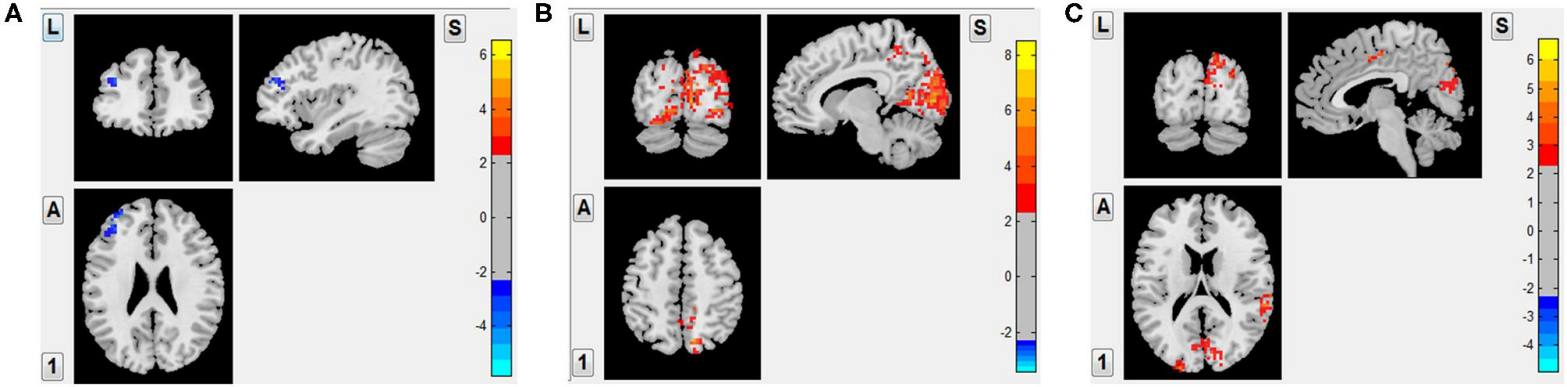
The alteration whole brain functional connectivity of 11 patients with MCS after different tDCS protocols. **(A)**: 1-week sham tDCS vs. the baseline: decreased FC intensity in left middle frontal gyrus (*FDR corrected p* < 0.001); **(B)**: 2-week real tDCS vs. the baseline: increased FC intensity in right precuneus and the bilateral occipital lobe (*FDR corrected p* < 0.001); and **(C)**: 2-week real tDCS vs. 1-week sham-tDCS: bilateral supplementary motor area, right angular gyrus, and right superior temporal gyrus (*FDR corrected p* < 0.001).

To investigate the possible local and remote effects of tDCS in the brain of patients with MCS and to explore the pertinent mechanism, a set of ROIs related to consciousness circuitries was selected as the targets, including occipital lobe, precuneus, supplementary motor area, angular gyrus, superior temporal gyrus, and middle frontal gyrus. The FC intensity of left middle frontal gyrus significantly decreased the post-1-week sham tDCS compared with the baseline (FDR corrected *p* < 0.001), but other brain networks showed no significant changes. In contrast, FC intensity was significantly increased in right precuneus, which was nearby the stimulated site post-2-week real tDCS compared with the baseline (FDR corrected *p* < 0.001). Importantly, FC intensity of the bilateral occipital lobe, which was distant from the left DLPFC, was also significantly increased in post-2-week real tDCS compared with the baseline (FDR corrected *p* < 0.001). Also, an increased FC intensity was also observed not only in the nearby brain regions of the stimulated site (e.g., left supplementary motor area), but also in the distant brain regions (including the right supplementary motor area, right angular gyrus, and right superior temporal gyrus) post-2-week real tDCS compared with those post-1-week sham tDCS (FDR corrected *p* < 0.001) (Figure 2; Table 3).

**Table 3:** Brain regions with alteration of functional connectivity (FC) after different transcranial direct current stimulation (tDCS) protocols.

**Brain regions**	**L/R**	**Peak MNI coordinates (mm)**			**CLuster size (Voxels)**	***t*** **-values**	**Trends**
		**X**	**Y**	**Z**			
**1-week Sham-tDCS VS. baseline**							
Middle frontal gyrus	L	−33	51	24	34	−3.73	T1 < T0
**2-week real-tDCS VS. baseline**							
Occipital lobe	R	13	−92	9	990	6.1	T2 > T0
Precuneus	R	12	−75	48	59	7.59	T2 > T0
Occipital lobe	L	−9	−90	6	356	8.54	T2 > T0
**2-weeks real-tDCS vs. 1-week Sham-tDCS**							
Occipital lobe	R	30	−87	39	192	4.29	T2 > T1
SMA	R	9	−45	36	32	4.33	T2 > T1
Angular gyrus	R	54	−60	30	49	4.3	T2 > T1
Superior temporal gyrus	R	60	−36	18	79	4.16	T2 > T1
Occipital lobe	L	−21	−93	15	32	5.15	T2 > T1
SMA	L	0	3	48	46	4.59	T2 > T1

*1. FDR corrected p < 0.001, cluster size > 20; R, right; L, left; T0, baseline; T1, 1-week sham tDCS; T2, 2-week real tDCS; SMA, supplementary motor area*.

## Discussion

The findings from this study demonstrated the effect of tDCS in treating patients with MCS and explored its possible mechanism. We found the increase of CRS-R total scores and alterations of FC in rs-fMRI mediated by 2-week real tDCS in patients with MCS. Although bias may be induced due to the lack of the control group; the recovery signs of consciousness observed by comparing the clinical assessment and neuroimaging data at the baseline, post-1-week sham stimulation, and 2-week real stimulation strengthen our findings.

In this study, the behavioral assessment showed that the CRS-R total scores were improved post-real DCS compared with the scores at the baseline in patients with MCS. However, no improvement in CRS-R was observed post-sham stimulation compared with the baseline. These results were consistent with previous pieces of research (27, 28). Angelakis et al. reported the effects of 2-week tDCS over left DLPFC in patients with different degrees of consciousness disorders, in which all the patients with MCS showed increased CRS-R scores at the end of the treatment (29). Another study demonstrated that 10 tDCS sessions over precuneus could improve the signs of consciousness in patients with DoC represented by CRS-R total scores (22). In addition, the auditory and motor CRS-R subscales scores were significantly higher post-2-week real tDCS than both the baseline and sham tDCS (Table 2), from which we speculated that the potential of tDCS may be related to its effect on auditory and motor functions.

Although highlighted in clinical trials that a better clinical outcome could be achieved by tDCS in DoC, little is known about its beneficial impact on neural activity and related network (30, 31). In addition, neuroimaging and electrophysiological assessments are more sensitive to identify changes caused by tDCS than a behavioral outcome (32). The FC analysis in our study showed that there was no strengthened FC but a decreased FC in left middle frontal gyrus post-1-week sham tDCS compared with the baseline. However, in post-2-week real tDCS, FC of bilateral supplementary motor area, right angular gyrus, and right superior temporal gyrus were significantly enhanced compared with that of post-1-week sham tDCS. In addition, the bilateral occipital lobe as a key node in visual network and the right precuneus as one of the key nodes in default network were all significantly activated post 2 weeks of real tDCS compared with the baseline.

Neurophysiologic and functional neuroimaging studies indicated that the recovery of the consciousness requires the participation and coordination of different brain regions in cerebrum (33). A decreased functional connectivity was found in left and right default modes, executive control, auditory, and attention networks in patients with MCS (33, 34). Previous studies in healthy participants showed that tDCS over left DLPFC could increase the FC between the left DLPFC and bilateral parietal regions (5, 35). Our findings showed that an increased FC in sensorimotor network (bilateral supplementary motor area), frontal parietal network (right angular gyrus), and auditory network (right superior temporal gyrus) post-2-week real tDCS, which provided another piece of evidence that improvement of FC in both nearby and distant of the stimulated brain regions could be induced by tDCS in patients with MCS (5, 36–39). The results of FC from rs-fMRI in our study were also in accordance with the conclusion of previous studies using electrophysiological analysis (13, 40). As to the specific mechanism, we speculated that the anodal tDCS over the left DLPFC could simultaneously activate the stimulated brain region locally and its related brain regions distantly, owing to the residual capacity and neural plasticity in patients with MCS (36, 39).

The present study supports the effectiveness of using tDCS over the left DLPFC in treating patients with MCS. Improved CRS-R scores and enhanced FC were revealed in some patients. Our study also had some limitations. One was that the sample size was relatively small, which may reduce the statistical power. The other limitation was that no follow-up was done to assess potential long-term treatment effects. Another limitation was the lack of the control group. Although we only included the patients with same CRS-R scores on the 3 assessment sessions in 2 weeks before the enrollment, we could not completely rule out the effect of spontaneous recovery. As this could be a drawback for the study, we would carry out further randomized controlled clinical pieces of research to explore the immediate and long-term effect of tDCS in patients with MCS.

## Conclusion

The present study demonstrated the potential of tDCS in treating MCS. Signs of consciousness in MCS could be improved through tDCS over left DLPFC, as measured by CRS-R total scores. The FC based on rs-fMRI was significantly increased in the stimulation site (left DLPFC) and distant regions mediated by tDCS.

## Data Availability Statement

The original contributions presented in the study are included in the article/Supplementary Material, further inquiries can be directed to the corresponding author/s.

## Ethics Statement

The studies involving human participants were reviewed and approved by Guangdong 999 Brain Hospital. The patients/participants provided their written informed consent to participate in this study. Written informed consent was obtained from the individual(s) for the publication of any potentially identifiable images or data included in this article.

## Author Contributions

YP, JZ, YL, and TY designed this study. YP, JZ, XL, JD, SZ, JZ, HL, and XZ performed the experiments. Data was analyzed by YP, JZ, YL, and XW. YP, JZ, and XW wrote the manuscript. All authors contributed to the article and approved the submitted version.

## Funding

This work was supported by the National Natural Science Foundation of China (81974357), Guangdong Basic and Applied Basic Research Foundation (2019A1515012097), Yangzhou Science and Technology Development Plan Project (YZ2020201), and Huxin Fund of Jiangsu Key Laboratory of Zoonosis (HX2003).

## Conflict of Interest

The authors declare that the research was conducted in the absence of any commercial or financial relationships that could be construed as a potential conflict of interest.

## Publisher's Note

All claims expressed in this article are solely those of the authors and do not necessarily represent those of their affiliated organizations, or those of the publisher, the editors and the reviewers. Any product that may be evaluated in this article, or claim that may be made by its manufacturer, is not guaranteed or endorsed by the publisher.
