# Supplementary material for: Efficacy of Transcranial Direct Current Stimulation Over Dorsolateral Prefrontal Cortex in Patients With Minimally Conscious State
Source: Front Neurol. 2022 Feb 18;13:821286. doi: 10.3389/fneur.2022.821286 (PMC8894202; doi:10.3389/fneur.2022.821286)
Supplement: Supplementary file 1 [file Data_Sheet_1.docx]

Table S1. The peak x,y,z coordinates of each ROI

| Regions (left/right) | BA [ROI centered at x, y, z] |
| --- | --- |
| Precuneus | 7 [-9 -72 37] [10 -69 39] |
| Middle frontal gyrus | 10[-51 3 48] [31 45 26] |
| Supplemental motor area | 6 [-4 14 48] [4 14 48] |
| Angular gyrus | 39 [-31 -59 42] [30 -61 39] |
| Superior temporal gyrus | 41/42 [-44 -6 11] [44 -6 11] |
| Occipital lobe | 17 [-13 -85 6] [8 -82 6] |

Table S2. CRS-R total and subscales scores of the 11 patients with MCS at each time point.

| Patient | T0 | | | | | |  | T1 | | | | | |  | T2 | | | | | |
| --- | --- | --- | --- | --- | --- | --- | --- | --- | --- | --- | --- | --- | --- | --- | --- | --- | --- | --- | --- | --- |
|  | A1 | O | V | C | M | A2 |  | A1 | O | V | C | M | A2 |  | A1 | O | V | C | M | A2 |
| P1 | 2 | 2 | 1 | 1 | 2 | 2 |  | 2 | 2 | 1 | 1 | 2 | 2 |  | 3 | 2 | 2 | 1 | 5 | 3 |
| P2 | 3 | 1 | 2 | 1 | 5 | 2 |  | 3 | 1 | 2 | 1 | 5 | 2 |  | 3 | 1 | 2 | 1 | 5 | 2 |
| P3 | 2 | 2 | 2 | 1 | 3 | 2 |  | 2 | 2 | 2 | 1 | 3 | 2 |  | 3 | 2 | 3 | 1 | 6 | 2 |
| P4 | 2 | 1 | 1 | 1 | 2 | 2 |  | 2 | 1 | 1 | 1 | 2 | 2 |  | 3 | 2 | 1 | 2 | 4 | 2 |
| P5 | 3 | 1 | 2 | 1 | 5 | 3 |  | 3 | 1 | 2 | 1 | 5 | 3 |  | 3 | 1 | 2 | 1 | 5 | 3 |
| P6 | 1 | 2 | 1 | 1 | 2 | 2 |  | 1 | 2 | 1 | 1 | 2 | 2 |  | 4 | 2 | 2 | 1 | 5 | 2 |
| P7 | 2 | 1 | 2 | 1 | 5 | 2 |  | 2 | 1 | 2 | 1 | 5 | 2 |  | 2 | 1 | 2 | 1 | 5 | 2 |
| P8 | 3 | 1 | 2 | 1 | 4 | 2 |  | 3 | 1 | 2 | 1 | 4 | 2 |  | 3 | 1 | 2 | 1 | 4 | 2 |
| P9 | 2 | 1 | 2 | 1 | 2 | 2 |  | 2 | 1 | 2 | 1 | 2 | 2 |  | 3 | 2 | 3 | 1 | 5 | 2 |
| P10 | 2 | 1 | 2 | 1 | 5 | 2 |  | 2 | 1 | 2 | 1 | 5 | 2 |  | 2 | 1 | 2 | 1 | 5 | 2 |
| P11 | 3 | 2 | 2 | 1 | 4 | 2 |  | 3 | 2 | 2 | 1 | 4 | 2 |  | 3 | 2 | 2 | 1 | 4 | 2 |

A1: Auditory; O: Oromotor; V: Visual; C: Communication; M: Motor; A2: Arousal.
